# An Examination of the Relationships between Eating-Disorder Symptoms, Difficulties with Emotion Regulation, and Mental Health in People with Binge Eating Disorder

**DOI:** 10.3390/bs13030234

**Published:** 2023-03-07

**Authors:** Felipe Q. da Luz, Mohammed Mohsin, Tatiana A. Jana, Leticia S. Marinho, Edilaine dos Santos, Isabella Lobo, Luisa Pascoareli, Tamiris Gaeta, Silvia Ferrari, Paula C. Teixeira, Táki Cordás, Phillipa Hay

**Affiliations:** 1 Eating Disorders Program (AMBULIM), Institute of Psychiatry, Faculty of Medicine, University of São Paulo, São Paulo 05403-010, SP, Brazil; 2 Sydney Medical School, Faculty of Medicine and Health, The University of Sydney, Sydney, NSW 2006, Australia; 3 Mental Health Research Unit, Liverpool Hospital, South Western Sydney Local Health District, NSW Health, Sydney, NSW 2170, Australia; 4 School of Clinical Medicine, Faculty of Medicine & Health, University of New South Wales, Sydney, NSW 2052, Australia; 5 Department of Neurosciences and Behavior, Institute of Psychology, University of São Paulo, São Paulo 05508-030, SP, Brazil; 6 Translational Health Research Institute, School of Medicine, Western Sydney University, Campbelltown, NSW 2560, Australia

**Keywords:** binge eating disorder, eating disorder, emotion regulation, mental health

## Abstract

Eating disorders, such as binge eating disorder, are commonly associated with difficulties with emotion regulation and mental-health complications. However, the relationship between eating-disorder symptoms, difficulties with emotion regulation, and mental health in people with binge eating disorder is unclear. Thus, we investigated associations between eating-disorder symptoms, difficulties with emotion regulation, and mental health in 119 adults with binge eating disorder. Participants were assessed with the Eating Disorder Examination Questionnaire, Loss of Control over Eating Scale, Difficulties in Emotion Regulation Scale, Depression Anxiety and Stress Scale, and the 12-Item Short Form Survey at the pre-treatment phase of a randomized controlled trial. Structural-equation-modelling path analysis was used to investigate relationships between variables. We found that (1) eating-disorder behaviors had a direct association with depression, anxiety, and stress; (2) depression, psychological stress, difficulties with emotion regulation, and eating-disorder psychopathology had a direct association with mental-health-related quality of life; and (3) eating-disorder psychopathology/behaviors and stress had a direct association with difficulties with emotion regulation. Our findings show that depression, stress, difficulties with emotion regulation, and eating-disorder psychopathology were related in important ways to mental-health complications in people with binge eating disorder.

## 1. Introduction

Binge eating disorder (BED) is an eating disorder characterized in the *Diagnostic and Statistical Manual of Mental Disorders 5* (*DSM-5*) by recurrent binge-eating episodes that have occurred at least once a week for the past three months [1]. Binge-eating episodes are defined as the ingestion of an amount of food that is larger than most people would consume under similar circumstances, accompanied with a sense of loss of control over eating [1]. The *DSM-5* criteria for BED also require that people experience at least three of the following five features: (1) eating much more quickly than normal; (2) eating until feeling excessively full; (3) overeating when not feeling physically hungry; (4) eating alone because of embarrassment related to the amount of food consumed; and (5) feeling disgusted, depressed or very guilty after binging, and reported marked distress with the binge eating [1].

People with BED commonly experience comorbid mental-health problems. For instance, a systematic review found that BED is significantly associated with depression [2], and another study reported that anxiety is an important factor in the development and maintenance of binge eating [3]. Moreover, a recent study in the United States found that BED was associated with lifetime mood disorders and anxiety disorders [4]. In addition to mood and anxiety disorders, psychological stress also has a relationship with the desire to binge eat in people with BED [5]. For instance, psychological stress can lead to a greater desire to binge eat in people with BED compared to those without BED [5]. Lastly, BED can also be associated with poor mental-health-related quality of life (HRQoL). For example, a study in Brazil found reduced mental HRQoL in people with BED compared to people without BED [6]. In line with this, another study found that people with obesity and comorbid BED experience poorer mental HRQoL in comparison to people with obesity but without BED, or people without obesity and without BED [7]. Moreover, mental HRQoL can be particularly poorer in women with BED compared to men with BED [7]. Taken together, the aforementioned studies showed significant relationships between BED and poor mental health (i.e., depression, anxiety, psychological stress, and reduced mental HRQoL).

In addition to poor mental health, people with eating disorders can experience difficulties with emotion regulation. Effective emotion regulation is the awareness, understanding, and acceptance of emotions, modulation of emotional arousal, and the ability to act in desired ways regardless of emotional state [8]. Emotional regulation is important for mental health, and ineffective emotion regulation can be an important factor in the maintenance of eating-disorder behaviors [9]. It is noteworthy that people with eating disorders report overall poorer emotion regulation in comparison to people without eating disorders [10], and that poor emotional awareness and clarity has been found in people with eating disorders [11]. Studies with samples of people with BED found similar results [12,13]. For instance, limited access to emotion-regulation strategies were associated with BED in people with obesity who were candidates for bariatric surgery [12]. Moreover, women with BED can experience greater emotion-regulation difficulties, namely nonacceptance of emotional responses, lack of emotional clarity, difficulties engaging in goal-directed behavior, impulse-control difficulties, and limited access to emotion-regulation strategies in comparison to women without BED [13]. Women with BED may also use more maladaptive emotion-regulation strategies (i.e., rumination, self-blame), and use less adaptive emotion-regulation strategies (i.e., positive refocusing, putting into perspective), in comparison to women without BED [13].

The existing literature indicates that there is an elevated occurrence of difficulties with emotion-regulation and mental-health problems in people with eating disorders such as BED. However, more research in this field is necessary, as difficulties with emotion regulation can be a risk factor for severe mental-health complications (e.g., suicidality) in people with eating disorders [14]. Thus, it is important to investigate the relationship between eating-disorder symptoms, difficulties with emotion regulation, and mental health, to potentially improve prevention and treatment programs for people with BED. To the best of our knowledge, no previous study thoroughly investigated the relationships between multiple measures of eating-disorder symptoms and mental health in people with BED using an advanced statistical analytic tool (e.g., structural-equation-modelling technique) to examine complex models. Thus, in this study we examined the relationships between eating-disorder psychopathology (i.e., dietary restraint, concerns about body shape, weight, and eating), eating-disorder behaviors (i.e., objective binge-eating episodes, subjective binge-eating episodes, loss of control over eating), difficulties with emotion regulation, poor mental health (i.e., symptoms of depression, anxiety, psychological stress), and mental HRQoL in a sample of adults with BED.

## 2. Materials and Methods

### 2.1. Study Design and Participants

We assessed data from pre-treatment measures of participants of a randomized controlled trial that investigated the efficacy of two different online treatment programs for people with BED and comorbid overweight or obesity [15]. Participants’ inclusion criteria were (1) age > 18 years; (2) BED, according to the DSM 5 criteria [1]; (3) body mass index (BMI) > 27 and <45 kg/m^2^; (4) being literate; (5) access to a computer with internet; (6) access to a private room to participate in the online therapy sessions; (7) time available to participate in the whole program; and (8) access to a scale and stadiometer to measure their body weight and height. Exclusion criteria were (1) having bariatric surgery in the previous 24 months; (2) simultaneous participation in another treatment for weight loss or binge eating; (3) clinical conditions that interfere with weight control (e.g., Prader–Willi syndrome, Cushing’s syndrome); (4) being pregnant; and (5) severe psychiatric disorder (i.e., schizophrenia, bipolar disorder) or a high suicide risk.

Recruitment of participants from the general community occurred via advertisements on the University of São Paulo’s social media from August 2020 to June 2022. The advertisement indicated that the research project offered online group therapy for people with BED and comorbid overweight or obesity, and included a link to a survey that could be completed by people that were interested in participating in the randomized controlled trial. This link led to an online screening survey with questions that assessed the inclusion/exclusion criteria, demographic characteristics, and contact information. Potentially eligible participants were invited for a semi-structured clinical interview via videoconference with a member of the research team. The interviewers assessed whether participants met the DSM-5 criteria for BED described in Table 1. Participants were also required to measure their body weight and height before the interview, and to provide this information to interviewers.

### 2.2. Ethics

The study was approved by the Research Ethics Committee of the University of São Paulo’s Faculty of Medicine Hospital (CAAE: 19551419.1.0000.0068) in Brazil.

### 2.3. Measures

#### 2.3.1. Demographic Characteristics

A self-report questionnaire was used to collect information on age, sex, race, body weight, height, occupation, marital status, and income.

#### 2.3.2. Eating Disorder Examination Questionnaire (EDE-Q)

The EDE-Q is a widely used 28-item self-report questionnaire derived from the “gold standard” interview for the assessments of eating disorders, namely the Eating Disorder Examination [16]. The EDE-Q was used to assess the quantity of objective and subjective binge-eating episodes, as well as the severity of eating-disorder psychopathology in the past 28 days. The EDE-Q generates a global score that is obtained by averaging the subscales (i.e., dietary-restraint, weight-concern, shape-concern, and eating-concern) scores, with higher scores indicating greater eating-disorder psychopathology. We used participants’ EDE-Q global scores to assess the severity of eating-disorder psychopathology in our study. Overall, the EDE-Q is a reliable and valid measure of eating-disorders symptoms [17]. We used an unpublished Brazilian-Portuguese version of the EDE-Q that was adapted from the European Portuguese EDE-Q and was previously used in research in Brazil [18,19]. In this study sample, Cronbach’s alpha (α) for the item pool of EDE-Q global score was 0.70.

#### 2.3.3. Loss of Control over Eating Scale (LOCES)

The experience of loss of control over eating constitutes a clinically significant feature of eating disorders. However, this feature is assessed only in a dichotomous “yes or no” manner in the EDE-Q, and this may lead to imprecise assessments. Therefore, the LOCES was used in the current study to complement assessments from the EDE-Q. The LOCES is a 24-item self-report scale that is used to assess the severity of a core feature of eating disorders, namely the loss of control over eating [20]. Each item is rated on a 5-point Likert scale that ranges from 1 (“never”) to 5 (“always”), which is averaged to generate a total score. Higher score on the LOCES indicate more severe loss of control over eating in the past 28 days. The LOCES shows good internal consistency and test–retest reliability, as well as convergent and discriminant validity [20]. We used a Brazilian-Portuguese version of the LOCES to assess loss of control over eating [21]. Cronbach’s alpha for the item pool of LOCES in this study was 0.91.

#### 2.3.4. Difficulties in Emotion Regulation Scale (DERS)

The DERS is a 36-item self-report scale that is widely used to assess clinically relevant difficulties in emotion regulation [8]. The DERS is used to assess the following 6 dimensions of difficulties with emotion regulation: lack of awareness of emotional responses, lack of clarity of emotional responses, non-acceptance of emotional responses, limited access to emotion-regulation strategies perceived as effective, difficulties controlling impulses when experiencing negative emotions, and difficulties engaging in goal-directed behaviors when experiencing negative emotions [8]. Each item is rated on a 5-point Likert scale of 1 (“almost never”) to 5 (“almost always”). For this study, we used only the total score of all 36 items, with higher scores indicating increased difficulties with emotion regulation. The DERS shows good construct validity, good internal consistency, and good discriminative ability [22]. The Brazilian-Portuguese version of the DERS was used in our study [23]. In this study sample, Cronbach’s alpha (α) for the total item pool of DERS was 0.86.

#### 2.3.5. Depression, Anxiety and Stress Scale (DASS-21)

The DASS-21 is a self-report scale with 21 items that is used to assess the magnitude of symptoms of depression (7 items), anxiety (7 items), and psychological stress (7 items) in both clinical and non-clinical samples [24]. Each item is rated on a 4-point Likert scale from 0 (“did not apply to me at all”) to 3 (“applied to me very much or most of the time”) assessing the severity of symptoms over the past week. For this study, the subscale scores were used separately with higher scores indicating more severe symptoms of depression, anxiety, or psychological stress. The DASS-21 is a valid measure of dimensions of depression, anxiety, and psychological stress, and shows appropriate construct validity and high reliability [25]. We used the Brazilian-Portuguese-validated version of the DASS-21 in our study [26]. Cronbach’s α for our sample was 0.92 for the total DASS-21 item pool, 0.89 for the depression subscale, 0.77 for the anxiety subscale, and 0.81 for the psychological stress subscale.

#### 2.3.6. 12-Item Short Form Survey (SF-12)

The SF-12 is a reliable measure used to assess mental and physical HRQoL in different population groups [27]. The SF-12 is also a valid and sensitive measure of impairment in HRQoL in people with eating disorders [28]. The survey scores are categorized into two domains, a physical-composite-scale (PCS) score and a mental-composite-scale (MCS) score, each including six items. In our study we analyzed only mental HRQoL using the MCS score. Elevated scores on the MCS indicate greater mental HRQoL. We used a Brazilian-Portuguese version of the SF-12 to assess participants’ mental HRQoL [29]. Cronbach’s alpha (α) for the MCS item pool was 0.70.

### 2.4. Statistical Analyses

Firstly, we documented the descriptive data for demographic characteristics (i.e., age, gender, race, occupation, marital status, income) and clinical features (i.e., eating-disorder psychopathology, objective binge-eating episodes, subjective binge-eating episodes, loss of control over eating, difficulties with emotion regulation, depression, anxiety, psychological stress, and mental HRQoL. Continuous variables were presented as means and standard deviation (SD); and categorical variables were presented as percentages. Next, we examined the associations of demographic characteristics with mean scores for all clinical features. We calculated a correlation matrix to explore potential correlations among clinical features. Theoretically relevant indices that showed a significant (*p* < 0.05) bivariate relationship with any of the clinical features were entered into the path model within a structural-equation-modelling (SEM) framework [30,31]. The SEM was designed to test the following associations: (1) inter-relationships among clinical features; (2) paths leading from objective or subjective binge-eating episodes, eating-disorder psychopathology, loss of control over eating, psychological stress, and anxiety, to difficulties with emotion dysregulation, depression, and mental HRQoL; (3) direct and indirect paths leading from binge eating, eating-disorder psychopathology, loss of control over eating, psychological stress, difficulties with emotion regulation, and depression, to mental HRQoL. Model fitness was assessed according to conventional criteria, including a non-significant chi-square test; comparative fit index (CFI) > 0.90; the Tucker–Lewis Index (TLI) > 0.90; the root-mean-square error of approximation (RMSEA) < 0.08; and the standardized root-mean-square residual (SRMR) < 0.08 [32,33,34]. The analyses were performed in SPSS v. 27 [35] and Mplus 7.1. [33].

## 3. Results

### 3.1. Participants’ Demographic Characteristics

One hundred and nineteen participants were included in our study (see Appendix A). The demographic characteristics of all 119 participants are shown in Table 2. The participants’ mean age was 36 years (SD, 8.8); 21.8% (n = 26) were 18 to 29 years of age, 45.4% (n = 54) were 30–39 years of age, and 32.8% (n = 39) were 40–59 years of age. Most participants were female (n = 108, 90.8%). Three quarters of the participants were from a white ethnicity group and the remainder (25%) consisted of black or other ethnic backgrounds. Almost two-thirds (66%) of the participants were full-time or part-time employed, and a similar proportion (65%) reported to be either married or living with a partner (see Table 2).

The mean scores of the clinical features were: 14.2 (SD, 15.2) for objective binge-eating episodes; 10.5 (SD, 8.0) for subjective binge-eating episodes; 3.75 (SD, 0.9) for eating-disorder psychopathology, 81.9 (SD, 14.1) for loss of control over eating; 100.3 (SD, 24.8) for difficulties with emotion regulation; 15.6 (SD, 9.7) for depression; 10.3 (SD, 7.4) for anxiety; 20.8 (SD, 8.3) for psychological stress; and 32.7 (SD, 9.7) for mental HRQoL (see Table 2 and Appendix B).

### 3.2. Bivariate Analyses

Association of participant’s demographic characteristics and mean scores for all clinical features are shown in Table 1. There were no significant differences observed for any of the clinical features by participants’ demographic characteristics. Table 3 shows the correlation matrix of all clinical features.

### 3.3. Path Analysis

**Figure 1:**
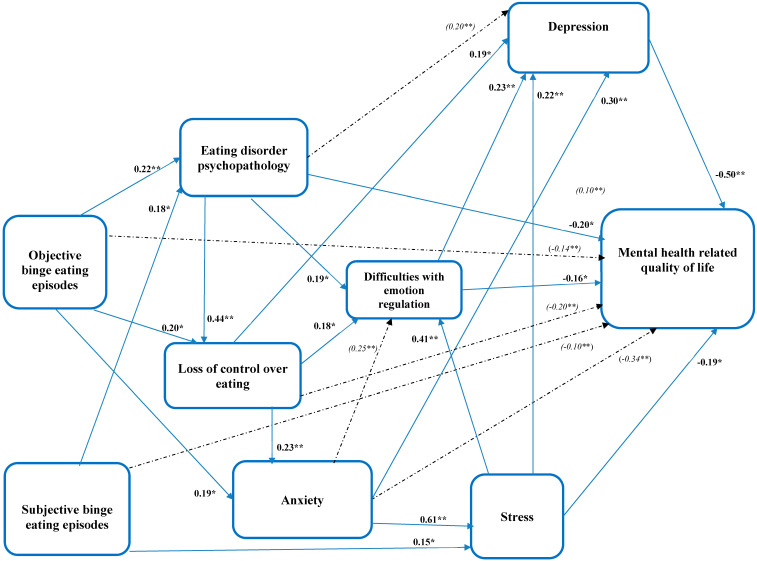
Path diagram: results from the structural-equation model, with standardized direct and indirect coefficients (β) of eating-disorder psychopathology, objective binge-eating episodes, subjective binge-eating episodes, loss of control over eating, anxiety, and psychological stress, to difficulties with emotion regulation, depression, and mental-health-related quality of life. Note. Significant indirect standardized coefficients are presented inside brackets (shown in italics). All variables included in the models are continuous, based on observed data, and no latent variables are used in the model. Dashed lines show significant indirect pathways. * Indicates significance level at *p* < 0.05; ** Indicates significance level at *p* < 0.01. Model summary: Chi-Square Test of Model Fit = 8.06, df = 15, *p* = 0.92: Comparative Fit Index (CFI) = 1.0; Tucker–Lewis Index (TLI) = 1.0; Standardized Root-Mean-Square Residual (SRMR) = 0.036; Root-Mean-Square Error of Approximation (RMSEA) = 0.001 (90%CI: 0.00–0.030).

Figure 1 displays the path diagram with standardized estimates (β) indicating direct and indirect associations. The model achieved a good fit with a non-significant chi-square value, χ^2^(15) = 18.06, *p* = 0.92; CFI = 1.00, TLI = 1.00, RMSEA < 0.001, and SRMR = 0.04.

#### 3.3.1. Correlates of Mental-Health-Related Quality of Life (HRQoL)

Depression (β = −0.50, *p* < 0.01), eating-disorder psychopathology (β = −0.20, *p* < 0.05), psychological stress (β = −0.19, *p* < 0.05), and difficulties with emotion regulation (β = −0.16, *p* < 0.05) showed significant direct associations with mental HRQoL (Figure 1). Indirect pathways to mental HRQoL included anxiety (indirect standardized coefficient = −0.34, *p* < 0.01) via psychological stress or depression; loss of control over eating (indirect standardized coefficient = −0.20; *p* < 0.001) via difficulties with emotion regulation or depression; objective binge-eating episodes (indirect standardized coefficient = −0.14; *p* < 0.01) via eating-disorder psychopathology; and subjective binge-eating episodes (indirect standardized coefficient = −0.10; *p* < 0.01) via eating-disorder psychopathology or psychological stress.

#### 3.3.2. Correlates of Difficulties with Emotion Regulation

Psychological stress (β = 0.41, *p* < 0.01), eating-disorder psychopathology (β = 0.19, *p* < 0.05), and loss of control over eating (β = 0.18, *p* < 0.05) showed significant direct associations with difficulties with emotion regulation (Figure 1). Additionally, we found an indirect pathway from anxiety to difficulties with emotion regulation via psychological stress (indirect standardized coefficient = 0.25, *p* < 0.01).

#### 3.3.3. Correlates of Depression

Loss of control over eating (β = 0.19, *p* < 0.05), anxiety (β = 0.30, *p* < 0.01), psychological stress (β = 0.22, *p* < 0.01), and difficulties with emotion regulation (β = 0.23, *p* < 0.01) showed significant direct associations with depression (Figure 1). Furthermore, eating-disorder psychopathology showed an indirect association with depression, via loss of control over eating and difficulties with emotion regulation (indirect standardized coefficient = −0.20, *p* < 0.01).

#### 3.3.4. Correlates of Anxiety

Objective binge-eating episodes (β = 0.19, *p* < 0.05) and loss of control over eating (β = 0.23, *p* < 0.01) showed significant direct associations with anxiety.

#### 3.3.5. Correlates of Psychological Stress

Subjective binge-eating episodes (β = 0.15, *p* < 0.05) and anxiety (β = 0.61, *p* < 0.01) showed significant direct associations with psychological stress.

#### 3.3.6. Correlates of Eating-Disorder Psychopathology

Objective binge-eating episodes (β = 0.22, *p* < 0.01) and subjective binge-eating episodes (β = 0.18, *p* < 0.05) showed significant direct associations with eating-disorder psychopathology.

#### 3.3.7. Correlates of Loss of Control over Eating

Objective binge-eating episodes (β = 0.20, *p* < 0.05) and eating-disorder psychopathology (β = 0.44, *p* < 0.01) showed significant direct associations with loss of control over eating.

## 4. Discussion

Our study investigated relationships between eating-disorder symptoms, difficulties with emotion regulation, general mental health, and mental HRQoL in adults with BED. Overall, we found that eating-disorder behaviors and psychopathology were associated with poorer mental health in participants included in our study. For instance, we found that: (1) objective binge eating had a direct association with anxiety; (2) subjective binge eating had a direct association with psychological stress; (3) loss of control over eating had a direct association with anxiety and depression; and (4) eating-disorder psychopathology had a direct association with mental HRQoL. Moreover, we found that eating-disorder psychopathology and loss of control over eating had a direct association with less effective emotion regulation. Lastly, we found that depression and psychological stress had a direct association with mental HRQoL, and psychological stress had a direct association with less effective emotion regulation.

Our study showed that several factors can be associated with poor mental HRQoL in people with BED. Our findings suggest that it is important to address a range of mental-health problems, i.e., depression, psychological stress, difficulties with emotion regulation, and eating-disorder psychopathology, to enhance mental HRQoL in this population. Thus, people with BED may require comprehensive assessment and treatment approaches—rather than treatments focused only on the cessation of binge-eating episodes—to improve their mental health. For instance, it is important that clinicians working with clients with BED assess their clients’ levels of depression, psychological stress, and difficulties with emotion regulation, and provide the required specialized therapies to address these complications when necessary. Clinicians can ask their clients with BED to complete self-report scales such as the DASS-21 and DERS to assess their mental-health status and difficulties with emotion regulation [8,24]. It may also be useful to comprehensively assess eating-disorder psychopathology in clients with BED, using measures such as the semi-structured interview for the investigation of eating-disorder symptoms, the Eating Disorder Examination [16]. This assessment can enable the identification of specific characteristics of the eating-disorder psychopathology that are prominent in each client, so that clinicians can address them and potentially prevent the deterioration of mental HRQoL. Moreover, it is noteworthy that some treatments for eating disorders can also induce improvements in general mental health. For instance, cognitive behavior therapy (CBT) for eating disorders can reduce depression, anxiety, mood intolerance, low self-esteem, clinical perfectionism, and interpersonal difficulties in people with BED [16,36,37].

Our findings also enable a better understanding of the occurrence of difficulties with emotion regulation in people with BED. We found that eating-disorder psychopathology, loss of control over eating, and psychological stress had a direct association with less effective emotion regulation in our sample of adults with BED. This finding suggests that a reduction in eating-disorder symptoms through CBT [38], and reduction in psychological stress via access to specialized treatments (e.g., mindfulness-based stress reduction) [39] may facilitate effective emotion regulation in people with BED. The attenuation of difficulties with emotion regulation is particularly important, as we found that such difficulties have a direct effect on depression and poor mental HRQoL in this population. Taking this into consideration, psychological therapies that focus on training in emotion-regulation skills (e.g., dialectical behavior therapy) are known to be useful to address mental-health complications and eating-disorder symptoms in people with BED and comorbid difficulties with emotion regulation [40], but are under researched [41,42]. Overall, it may be beneficial that clinicians working with treatment models that focus mostly on the reduction of eating-disorder symptoms consider adding skills training on emotion-regulation and stress-management interventions to their treatment plans for clients with BED.

In addition to direct relationships between eating-disorder symptoms, difficulties with emotion regulation, and mental health in people with BED, we also found significant indirect relationships. These indirect relationships were described in detail in the Results section; nonetheless, here we provide a summary: (1) objective and subjective binge eating, loss of control over eating, and anxiety showed an indirect association with mental-health-related quality of life; (2) eating-disorder psychopathology showed an indirect association with depression; and (3) anxiety showed an indirect association with difficulties with emotion regulation. We found multiple direct and indirect relationships between eating-disorder symptoms, difficulties with emotion regulation, and poor mental health in people with BED. Overall, the relationships that were found in our study provide a better understanding of the complexity of psychopathology associated with BED. However, our findings do not substitute individualized clinical assessments of symptoms of BED and associated mental-health complications. Clinicians working with clients with BED will need to conduct individual assessments of eating-disorder symptoms, difficulties with emotion regulation, and mental-health status, to understand how these factors influence each other in order to plan individualized treatments.

Our findings also have implications for research on treatment outcomes for people with BED. A significant number of treatment trials for BED focus on the reduction or abstinence of binge eating as an outcome, and neglect broader aspects of mental health and mental HRQoL [43]. This limits the understanding of the efficacy and effectiveness of treatments for BED. Thus, the inclusion of general measures of mental-health status (e.g., DASS-21 [24]) in treatment trials for people with BED is necessary to investigate potential effects of these treatments on overall mental health. Moreover, future research—including longitudinal studies—is necessary to elucidate causality, mediation, and bidirectional analyses between eating-disorder symptoms, difficulties with emotion regulation, and mental health in people with BED.

This study has several strengths and limitations. Notable strengths include the use of an advanced statistical analytical approach (i.e., the structural-equation-modelling technique)—to examine complex causal models. Additionally, we included several measures of eating-disorder symptoms (i.e., EDE-Q, LOCES) and aspects of mental health (i.e., DASS-21, DERS, SF-12 MCS score) in a sample of adults with BED. The combined use of these different statistical analyses and measures allowed us to examine relationships between eating-disorder symptoms, difficulties with emotion regulation, and mental health, in a comprehensive and reliable manner. The main limitation of our study is that we used a cross-sectional design, and causal inferences cannot be made. Another potential limitation is that 90.8% of the study sample was female. There were no significant differences between males and females in difficulties-with-emotion-regulation scores in our sample; however, our findings may not be generalizable to males with BED or people with BED in regions with significant cultural differences. Additionally, a potential limitation is that all data used for statistical analyses in this study were obtained via self-report measures. It is possible that we could have obtained more accurate clinical data if instead of self-report measures we used semi-structured interviews, such as the Eating Disorder Examination [16]. A final limitation of our study is that while we thoroughly examined effects of eating-disorder symptoms on mental-health status, we did not examine relationships in the opposite direction (i.e., potential effects of mental-health status on eating-disorder symptoms).

In summary, our study found multiple direct and indirect relationships between eating-disorder symptoms, difficulties with emotion regulation, and mental-health status in adults with BED. We found that depression, psychological stress, difficulties with emotion regulation, and eating-disorder psychopathology had a direct association with mental HRQoL. Additionally, eating-disorder psychopathology, loss of control over eating, and psychological stress had a direct association with difficulties with emotion regulation. Thus, research involving the evaluation of clinical-trial outcomes as well as real-world treatment plans for people with BED should address comprehensively the symptoms of depression, psychological stress, difficulties with emotion regulation, eating-disorder psychopathology (i.e., dietary restraint, excessive concerns about body shape, weight, and eating) and loss of control over eating.

## Figures and Tables

**Table 1:** *DSM-5* diagnostic criteria for binge eating disorder used in the present study.

Characteristics of a binge-eating episode:	- Eating, in a discrete period of time (e.g., within any 2-h period), an amount of food that is definitely larger than what most people would eat in a similar period of time under similar circumstances. - A sense of lack of control over eating during the episode (e.g., a feeling that one cannot stop eating or control what or how much one is eating).
Binge-eating episodes were associated with three or more of the following five criteria:	Eating much more rapidly than normal. Eating until feeling uncomfortably full. Eating large amounts of food when not feeling physically hungry. Eating alone because of feeling embarrassed by how much one is eating. Feeling disgusted with oneself, depressed, or very guilty afterward.
Additional criteria:	- Marked distress regarding binge eating. - Binge eating occurred at least once a week in the past 3 months. - Binge eating was not associated with recurrent use of inappropriate compensatory behavior (e.g., self-induced vomiting, misuse of laxatives/diuretics, fasting, excessive exercise).

Note. *DSM-5* = *Diagnostic and Statistical Manual of Mental Disorders 5th edition*.

**Table 2:** Participants’ demographic characteristics and associations with clinical features.

Demographic Characteristics		Mean Scores of Clinical Features								
		Difficulties with Emotion Regulation	Objective Binge Eating	Subjective Binge Eating	Eating-Disorder Psychopathology	Loss of Control over Eating	Depression	Anxiety	Stress	Mental-Health-Related Quality of Life
	Number (%)	Mean	Mean	Mean	Mean	Mean	Mean	Mean	Mean	Mean
All participants	119 (100.0)	100.3	14.2	10.5	3.7	81.9	15.6	10.3	20.8	32.7
Gender										
Male	11 (9.2)	100.4	19.8	10.3	3.6	82.8	16.2	10.9	24.4	29.0
Female	108 (90.8)	100.2	13.7	10.6	3.7	81.8	15.6	10.3	20.5	33.1
*p*-values from *t*-test		0.99	0.20	0.91	0.64	0.82	0.85	0.78	0.14	0.18
Age group										
<30	26 (21.8)	106.1	13.4	11.3	3.8	84.3	14.8	10.8	22.5	32.3
30–39	54 (45.4)	96.8	15.7	10.4	3.7	82.5	16.2	9.5	20.5	32.0
40 and above	39 (32.8)	101.1	12.8	10.2	3.7	79.5	15.5	11.1	20.3	34.0
*p*-values from F-test		0.28	0.63	084	0.81	0.37	0.83	0.53	0.52	0.60
Race										
White	89 (75.0)	99.3	13.1	10.6	3.7	82.1	15.7	10.6	21.0	33.3
Black and others	30 (25.0)	103.2	17.6	10.4	3.7	81.3	15.5	9.4	20.3	31.0
*p*-values from *t*-test		0.45	0.16	0.93	0.739	0.80	0.94	0.43	0.663	0.27
Occupation										
Employed full/part time	78 (66)	98.7	15.5	10.9	3.7	83.2	15.5	10.6	21.3	32.9
Unemployed/Others	41 (34)	103.1	11.8	9.8	3.7	79.4	16.0	9.8	20.0	32.5
*p*-values from *t*-test		0.36	0.20	0.44	0.90	0.16	0.81	0.55	0.40	0.82
Marital status										
Married/Living with partner	77 (65)	100.8	15.7	10.7	3.8	83.3	15.4	10.1	21.0	33.1
Single/Never married/Others	42 (35)	99.3	11.5	10.2	3.6	79.4	16.1	10.8	20.5	32.0
*p*-values from *t*-test		0.75	0.15	0.71	0.34	0.15	0.68	0.63	0.76	0.53
Income										
None to BRL 3.135	69 (58)	99.6	14.3	10.4	3.7	81.9	15.4	10.5	20.2	33.3
Above BRL 3.135	50 (42)	101.1	14.1	10.7	3.6	82.0	15.9	10.1	21.8	31.9
*p*-values from *t*-test		0.74	0.95	0.83	0.47	0.96	0.80	0.76	0.30	0.42

Note. BRL = Brazilian Real.

**Table 3:** Correlation Matrix: Correlations (associations) coefficients among all clinical features.

	Measures and Correlation Co-Efficient (*r*)								
Measures	Difficulties with Emotion Regulation	Objective Binge Eating	Subjective Binge Eating	Eating-Disorder Psycho-Pathology	Loss of Control over Eating	Depression	Anxiety	Stress	Mental-Health-Related Quality of Life
	*r*	*r*	*r*	*r*	*r*	*r*	*r*	*r*	*r*
Difficulties with emotion regulation	1.00								
Objective binge-eating episodes	0.09	1.00							
Subjective binge-eating episodes	0.09	0.07	1.00						
Eating-disorder psychopathology	0.34 **	0.21 *	0.19 *	1.00					
Loss of control over eating	0.31 **	0.29 **	0.13	0.48 **	1.00				
Depression	0.49 **	0.07	0.06	0.27 **	0.32 **	1.00			
Anxiety	0.34 **	0.23 *	−0.01	0.17	0.28 **	0.52 **	1.00		
Stress	0.47 **	0.07	0.16	0.21 *	0.16	0.57 **	0.63 **	1.00	
Mental-health-related quality of life	−0.47 **	0.00	−0.13	−0.33 **	−0.25 **	−0.67 **	−0.35 **	−0.49 **	1.00

* *p* < 0.05; ** *p* < 0.01.

**Figure A1:**
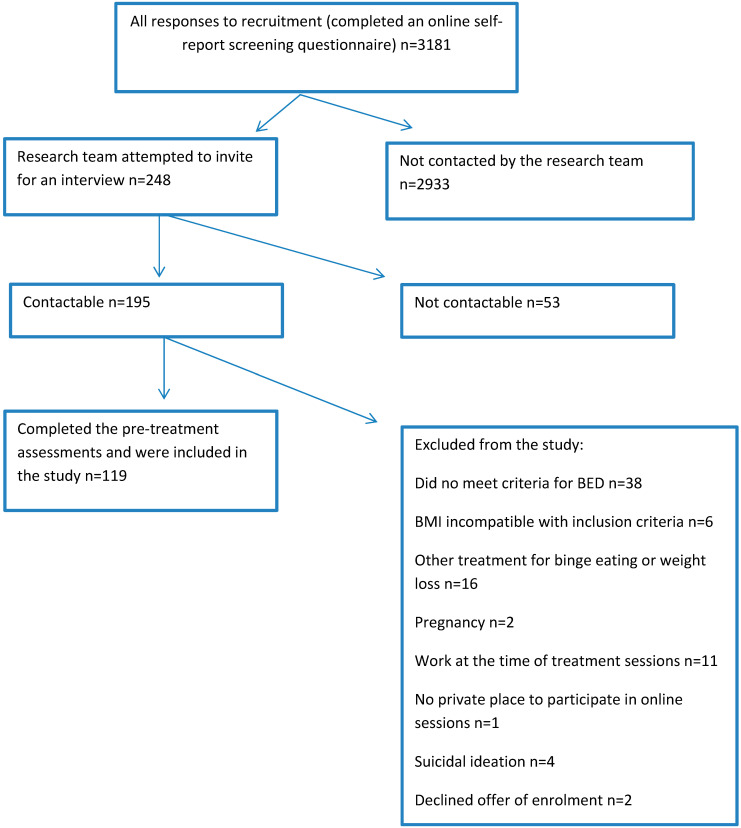
Participant flow.

## Data Availability

Data available on request, due to restrictions. The data presented in this study are available on request from the corresponding author. The data are not publicly available, due to privacy reasons.

## Appendix B

**Table A1:** Mean and standard deviation (SD) with 95% confidence interval (CI) and range for all clinical features.

		95% CI for Mean	Range
Clinical Features	Mean (SD)	(Lower Bound, Upper Bound)	Minimum, Maximum
Difficulties with emotion regulation	100.3 (24.8)	(95.8, 100.8)	47.0, 161.0
Objective binge-eating episodes	14.2 (15.2)	(11.5, 17.0)	0.0, 151.0
Subjective binge-eating episodes	10.5 (8.0)	(9.1, 12.0)	0.0, 28.0
Eating-disorder psychopathology	3.7 (0.9)	(3.5, 3.9)	1.9, 5.5
Loss of control over eating	81.9 (14.1)	(79.4, 84.5)	42.0, 115.0
Depression	15.6 (9.7)	(13.9, 17.4)	0.0, 40.0
Anxiety	10.3 (7.4)	(9.0, 11.7)	0.0, 30.0
Stress	20.8 (8.3)	(19.3, 22.3)	2.0, 38.0
Mental-health-related quality of life	32.7 (9.7)	(31.0, 34.5)	13.3, 55.9
